# Two Different Methods to Measure the Stability of Acetabular Implants: A Comparison Using Artificial Acetabular Models

**DOI:** 10.3390/s20010254

**Published:** 2020-01-01

**Authors:** Quentin Goossens, Leonard Cezar Pastrav, Michiel Mulier, Wim Desmet, Jos Vander Sloten, Kathleen Denis

**Affiliations:** 1 Department of Mechanical Engineering, Campus Group T, KU Leuven, 3000 Leuven, Belgium; 2 Department of Orthopedics, University Hospital Leuven, 3000 Leuven, Belgium; 3 Department of Mechanical Engineering, PMA Division, KU Leuven, 3000 Leuven, Belgium; 4 Department of Mechanical Engineering, Biomechanics Section, KU Leuven, 3000 Leuven, Belgium

**Keywords:** total hip arthroplasty, acetabular implant stability, micromotion

## Abstract

The total number of total hip arthroplasties is increasing every year, and approximately 10% of these surgeries are revisions. New implant design and surgical techniques are evolving quickly and demand accurate preclinical evaluation. The initial stability of cementless implants is one of the main concerns of these preclinical evaluations. A broad range of initial stability test methods is currently used, which can be categorized into two main groups: Load-to-failure tests and relative micromotion measurements. Measuring relative micromotion between implant and bone is recognized as the golden standard for implant stability testing as this micromotion is directly linked to the long-term fixation of cementless implants. However, specific custom-made set-ups are required to measure this micromotion, with the result that numerous studies opt to perform more straightforward load-to-failure tests. A custom-made micromotion test set-up for artificial acetabular bone models was developed and used to compare load-to-failure (implant push-out test) with micromotion and to assess the influence of bone material properties and press-fit on the implant stability. The results showed a high degree of correlation between micromotion and load-to-failure stability metrics, which indicates that load-to-failure stability tests can be an appropriate estimator of the primary stability of acetabular implants. Nevertheless, micromotions still apply as the golden standard and are preferred when high accuracy is necessary. Higher bone density resulted in an increase in implant stability. An increase of press-fit from 0.7 mm to 1.2 mm did not significantly increase implant stability.

## 1. Introduction

Cementless fixation is currently the most used technique for acetabular implants [1]. The initial stability of cementless THA prostheses is obtained during the intra-operative placement of the implant by means of a press-fit fixation into the host bone. This initial stability can be quantified by the reversible relative motion between implant and host bone under physiological loading immediately after implantation, also denoted as micromotion. This relative motion is one of the main factors in determining proper osseointegration of the implant over time [2], and so enabling a long lifetime of the implant. In order to enable this osseointegration, no more than 150 µm relative motion between implant and bone should occur at the interface under short term post-operative physiological loading [3,4]. The possibility to measure these micromotions in vitro can provide valuable information for new orthopedic implant designs, new surgical procedure development, or to validate implant stability monitoring methods, such as vibration analysis [5].

Several studies investigated the micromotion of cementless acetabular implants in replicate or cadaveric bone models by using specific custom-made set-ups. The use of linear variable differential transducers (LVDT) is a common measurement implementation amongst these studies [6,7,8,9,10]. Other implementations are eddy current displacement sensors [11,12] and optical measurements [13]. Measuring the micromotion can be challenging as specific custom-made set-ups need to be available. As an alternative to micromotion measurements, load-to-failure stability tests are widely used as these are easily conducted in universal testing machines and require less advanced set-ups. Commonly used load-to-failure tests in the literature are: Push/pull-out [14,15], lever-out [14,15,16,17,18], torsional loading [14,15,17,19,20] or edge loading [21] experiments. Most of these tests use stability metrics derived from the force-displacement curve. Combinations of micromotion measurements with the previously mentioned load-to-failure tests are also applied in studies. During these combined tests, the implant is loaded until a predefined threshold of micromotion at the bone-implant interface is reached. The load that corresponds to this micromotion threshold is then used as a stability metric [22,23,24,25].

The purpose of this study is to investigate the relationship between relative bone-implant micromotions which are widely accepted as the golden standard for primary orthopedic implant stability and the more traditional and commonly used load-to-failure implant stability metrics derived from a push-out experiment. As far as the authors know, this is the first implant stability study that quantifies the relationship between micromotion measurements and load-to-failure tests of cementless acetabular implants. Secondly, the influence of different variables, such as bone material properties and applied press-fits on the micromotions and push-out stability metrics is studied.

## 2. Materials and Methods

### 2.1. Acetabular Models and Cementless Acetabular Implant

**Figure 1:**
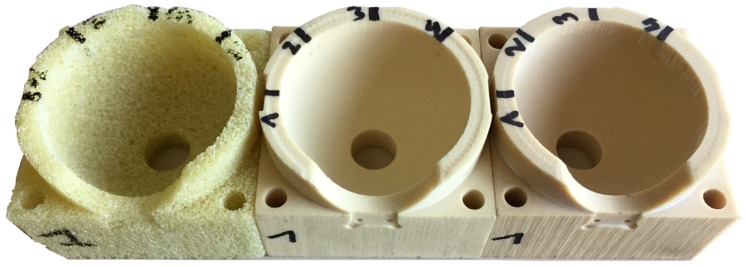
Acetabular models, including an acetabular notch made out of the three different replicate bone materials; rigid cellular foam 0.32 g/cc (**left**, denoted as C), solid rigid foam 0.48 g/cc (**middle**, denoted as L) and solid rigid foam 0.80 g/cc (**right**, denoted as H).

Acetabular models were custom-made from replicate bone material (Sawbones, Vashon Island, WA, USA) using a Computer Numerical Controlled (CNC) milling machine (Haas Automation Inc., Oxnard, CA, USA). CNC milling was preferred over surgical reaming to prepare the bone models in order to obtain well-controlled and repeatable acetabular cavity geometries. These replicate bone materials have similar mechanical properties as human trabecular bone [26], and are widely used in the literature to mimic real human bone in mechanical testing of the acetabulum [6,7,14,15,17,21,22,27]. Three different replicate bone materials were used to manufacture the acetabular models: A rigid cellular polyurethane foam with a density of 0.32 g/cc and a compressive modulus of 137 MPa (denoted as cellular) and two types of solid rigid polyurethane foam; one with a density of 0.48 g/cc and compressive modulus of 445 MPa (denoted as low density) and one with a density of 0.80 g/cc and compressive modulus of 1148 MPa (denoted as high density). A hemispherical cavity, including the acetabular rim with a discontinuity to represent the acetabular notch, was milled in each model. A hole with a diameter of 14 mm was drilled in de dome of the acetabular cavity in order to have access to the implant when performing the push-out test. Furthermore, four holes were drilled at the corners of the model in order to fixate the model in the test set-up. Figure 1 shows the geometry of the acetabular models. Two different cavity diameters were milled in the acetabular models (51.5 mm and 51.0 mm), to test the influence of different press-fits. A set of three identical bone model specimens was manufactured for each unique combination of density and cavity diameter, except for the 0.32 g/cc density model of which only one specimen per cavity diameter was manufactured. This resulted in fourteen acetabular bone model specimens divided over six different types as listed in Table 1.

**Figure 2:**
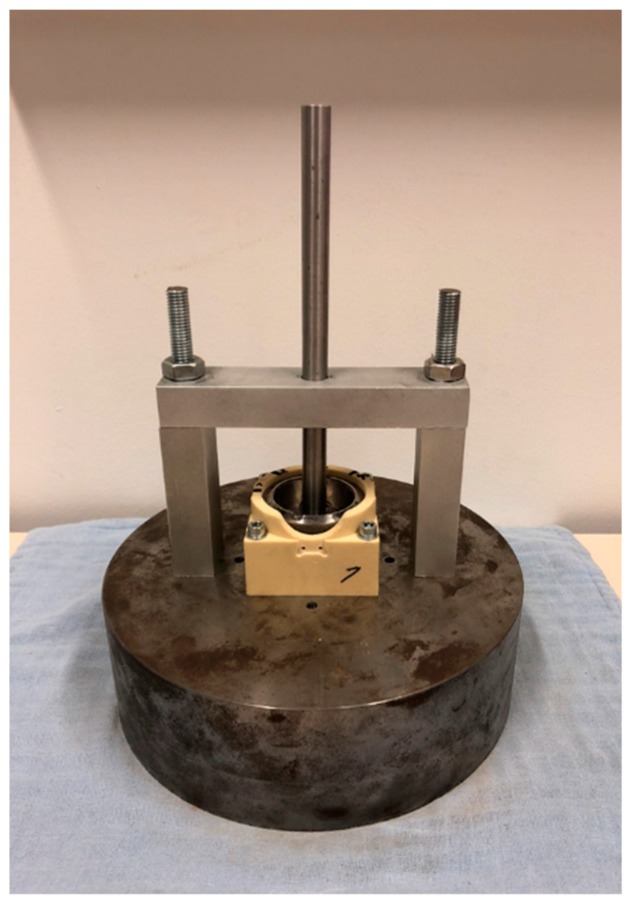
Guiding system to insert the implant in the acetabular models.

A Procotyl L cementless acetabular cup (Microport Orthopedics Inc., Arlington, TN, USA) with a diameter of 52.2 mm was used in combination with the acetabular models, resulting in a press-fit of 0.7 mm and 1.2 mm depending on the acetabular cavity diameter. Given the precisely manufactured acetabular cavity in this study, a maximal press fit of 1.2 mm was preferred to guarantee to complete seating of the cup, especially in the stiffest acetabular model (H510). Previous research showed that 1 mm press-fit in stiff polyurethane foam models or bovine bone models proved to be preferable over 2 mm in terms of implant stability and bone-implant contact area [17]. Clinical press-fits vary between 1 mm and 3 mm as they take in to account bone asperities and possible over reaming caused by user variability [28]. The acetabular cup was implanted in the acetabular models using hammer hits in combination with a guiding system (Figure 2). This guiding system allowed to guarantee identical cup orientation and seating for every tested specimen. Cup subsidence was measured during the insertion process using a caliper and full seating accorded to a convergence of the cup subsidence in the bone specimen. Additionally, the polar gap was inspected to be zero after insertion through the hole in the dome of the acetabular model. After the implant was inserted in the acetabular model, a micromotion measurement and push experiment were performed for every bone specimen. The entire protocol was repeated a second time for nine acetabular models (specimen 3, 4, 5, 7, 8, 10, 11, 12 and 13) to enlarge the sample size and to investigate the influence of reinserting the acetabular cup. The influence of this reinsertion on different stability metrics was statistically analyzed.

### 2.2. Implant Stability Testing

#### 2.2.1. Relative Micromotion

**Figure 3:**
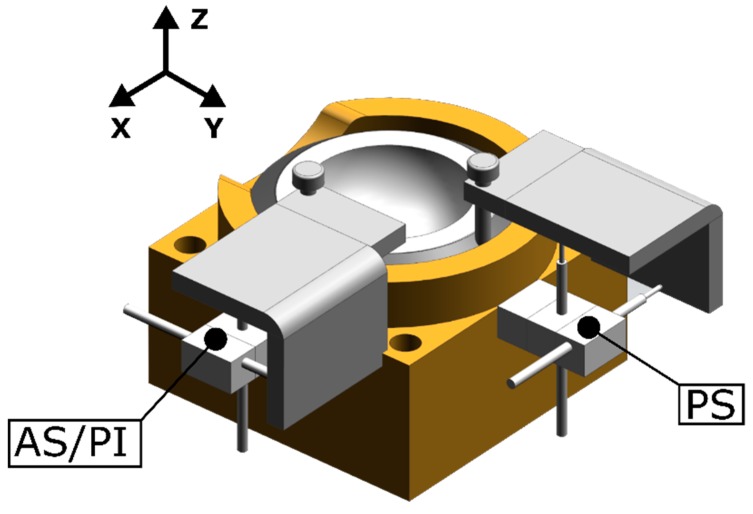
The positioning of the linear variable differential transducers (LVDT) sensors to measure the two-dimensional micromotion. The measurement locations corresponding to the anterior-superior (AS) or posterior-inferior (PI) zone and the posterior-superior (PS) zone of the acetabulum are indicated. The LVDT’s are oriented in z and y direction at the AS/PI location and z and x direction at the PS location according to the reference coordinate system.

Relative micromotion between the acetabular cup and host bone under physiological loading was measured for all specimen by four linear variable differential transducers (LVDT) with a resolution of 1 µm (GHSM-2.5B, Singer Instruments, Tirat Carmel, Israel) at a sample frequency of 50 Hz. Two measurement locations that correspond to the anterior-superior or posterior-inferior (according to the symmetry of the bone model) and posterior-superior zone of the acetabulum were included in this study. The two-dimensional relative micromotion was measured using two perpendicularly oriented LVDT’s in the plane parallel to the bone-implant interface at each measurement location. Figure 3 presents the measurement system. The LVDT housings (reference point) were rigidly connected to the acetabular model at 12 mm distance from the peripheral rim towards the dome of the acetabulum. The measuring probe of the LVDT’s was in contact with a compact plate structure that was rigidly connected to the acetabular cup (measuring point). The plate structure consisted out of two perpendicular surfaces which were aligned perpendicularly to the LVDT’s axes which allowed to measure the resulting two-dimensional translation of the plate relative to the acetabular bone model. This resulting local translation motion was used as an estimation of the true local micromotion at the bone-implant interface and was calculated using the following equations depending on the measurement locations;

(1) $$RMM_{AS/PI}=\sqrt{dy^{2}+dz^{2}}$$

(2) $$RMM_{PS}=\sqrt{dx^{2}+dz^{2}}$$

where *dx*, *dy* and *dz* are the local displacements measured by the LVDT’s according to their orientation.

Additionally, the total resulting micromotion (TRMM) was calculated as a single global metric for implant stability;

(3) $$TRMM=\sqrt{RMM_{AS/PI}{}^{2}+RMM_{PS}{}^{2}}$$

**Figure 4:**
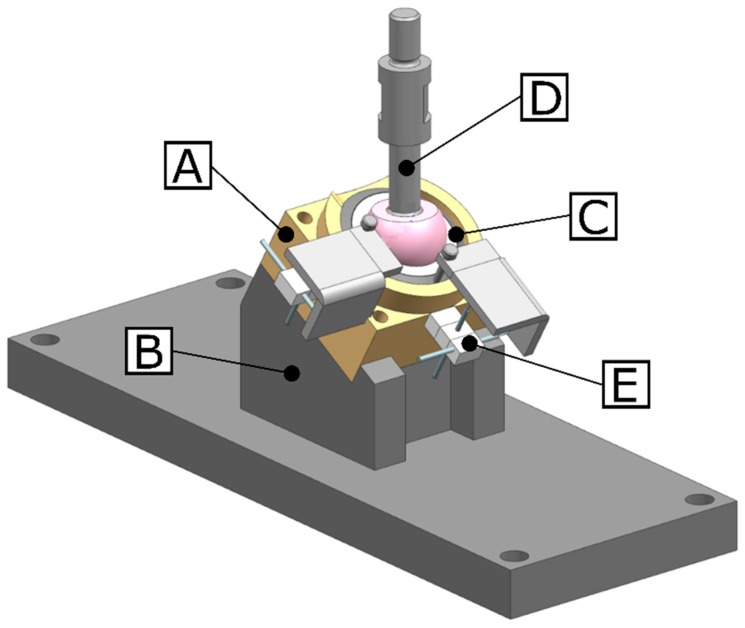
Micromotion test set-up as placed in the universal testing machine. In this presented set-up, the acetabular model (**A**) was mounted on a 30° inclined support block (**B**). The load was transmitted from the testing machine to the acetabular cup (**C**) through the femoral head connected to a custom-made rod (**D**). Relative motion between implant and bone was measured by the micromotion system (**E**).

Physiological loading conditions were applied to the implant-bone specimen using a universal testing machine (Instron 3369 Dual column table frame, Illinois Tool Works Inc., Norwood, MA, USA). The bone-implant system was positioned under 30° inclination, relative to the machine axis in order to mimic the coronal tilt of the acetabulum and obtain a physiologic loading orientation (Figure 4). In order to test the influence of the loading orientation, an additional horizontal measurement was performed in which the bone-implant set-up was positioned at 0° (acetabular plane oriented normal to the load vector). One hundred load cycles were applied to the bone-implant system through a custom-made rod with a 36 mm diameter titanium head articulating with a polyethylene liner in the acetabular cup. The load cycled between 0.1 kN and 2 kN at a velocity of 2 mm/s. The maximal applied load of 2 kN was chosen to correspond with the single leg stance joint load of a person of 80 kg [29].

#### 2.2.2. Push-Out Experiment

**Figure 5:**
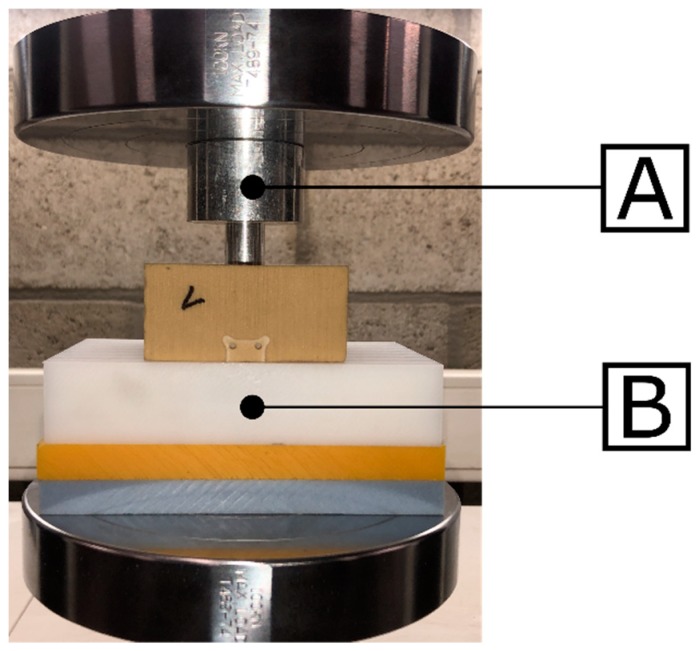
The set-up used for the push-out experiment. Two custom-made components were placed in the testing machine; a cylindrically shaped rod (**A**) to push the implant out of the acetabular model and a supporting component (**B**) that prevented only the acetabular model to move vertically. A cavity was provided in the supporting component to collect the acetabular cup when it was fully extracted out of the bone model.

**Figure 6:**
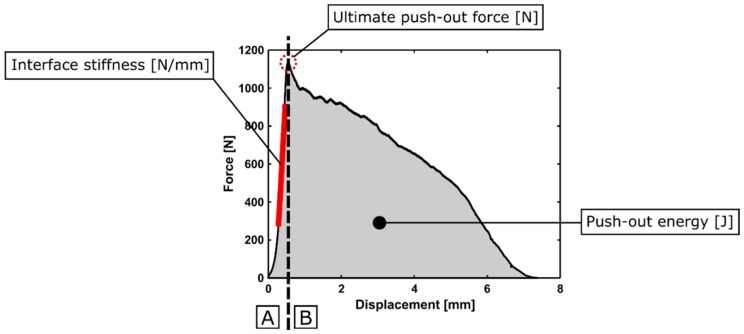
Typical force-displacement curve during a push-out experiment, including the different stability metrics (ultimate push-out force, interface stiffness and push-out energy). Two phases were distinguished: Elastic deformation of the bone model (**A**) and sliding of the cup relatively to the bone model (**B**).

After measuring the relative micromotion between the acetabular cup and bone specimen, the set-up was put upside down to allow the testing machine to push the implant out of the bone specimen. Two custom-made parts were used for this push-out experiment; a plastic supporting component for the acetabular model and a cylindrically shaped aluminum component to push the implant out of the acetabular model through the hole at the dome of the acetabulum (Figure 5). A constant displacement rate of 1 mm/min was applied, and the applied force and displacement were measured at a sample frequency of 10 Hz. As shown in Figure 6, the typical force-displacement curve consists out of two phases: A first linear phase during which an elastic deformation of the bone model is occurring whilst the cup does not move in relation to the acetabulum, and a second phase, during which the cup starts sliding relatively to acetabulum, and the force gradually decreases until the cup is fully extracted from the bone model. Three different stability metrics were calculated out of the force-displacement curve of the push-out experiment:

- Ultimate push-out force: The maximum force recorded during the push-out test.
- Interface stiffness: The positive slope of the force-displacement curve between 25% and 75% of the ultimate push-out force. The range of 25% to 75% was chosen as linear behavior was observed in this region of the force-displacement curve (Figure 6).
- Push-out energy: The area under the force-displacement curve of the push-out test.

The different stability metrics derived from an example of a force-displacement curve of a push-out experiment are shown in Figure 6.

### 2.3. Repeatability

Two acetabular bone models (L515 and H515) were used to assess the repeatability of the micromotion measurement set-up. The repeatability test consisted out of five times disassembling and re-assembling the entire measurement set-up followed by a micromotion measurement. The micromotion measurement was performed under 30° loading orientation as described earlier. The repeatability is reported as the standard deviations on the measured RMM at both measurement locations of the five repetitions.

### 2.4. Data Processing a Statistical Analysis

All data processing was performed using Matlab (The Mathworks, Natick, MA, USA). The statistical analysis was performed using IBM SPSS Statistics 25 (SPSS Inc., Chicago, IL, USA). Non-parametric tests were used due to the limited sample size. The Wilcoxon-Mann-Whitney test was used to compare the stability metrics (local RMM, TRMM, ultimate push-out force, interface stiffness and push-out energy) between the tested variables (densities and press-fits). The Wilcoxon signed rank test was used to assess the influence of reinserting the implant in the acetabular model and to assess the influence of the loading orientation. Initial sample size per group varied between one (C515 and C510) and three (L515, L510, H515 and H510). A linear regression analysis was used to assess the relationship between the different stability metrics. Nine bone specimens were retested to enlarge the sample size up to 23 for the linear regression analysis. Linear correlation coefficients (R^2^) were calculated between the different stability metrics. Significance was set at the five percent level for all tests.

## 3. Results

### 3.1. Repeatability and Influence of Reinserting the Acetabular Implant in the Acetabular Models

The micromotion measurement system showed to have a high degree of repeatability following an entire disassembly and re-assembly of the set-up. The standard deviation on the local RMM of the H510 and L515 specimen was 1.17 µm and 2.33 µm at the AS/PI zone and 2.03 µm and 2.38 µm at the PS zone, respectively. Reinsertion of the acetabular implant in the acetabular models did not lead to significant differences in local RMM or TRRM (*p*-Values varied between 0.069 and 0.779). This observation made it possible to group the micromotion results of the first and second insertion to maximize the sample size per acetabular model up to 6. A significant difference was observed in the results of the push-out experiments between the first and second insertion of the acetabular model. Push-out force (*p* = 0.015) and interface stiffness (*p* = 0.021) showed to be significant lower (13.3% and 11.9%, respectively) after the second insertion of the acetabular implant. No significant difference in push-out energy was observed (*p* = 0.110). Due to these observations, it was chosen to only consider the results of the push-out stability tests after the first implant insertion. The sample sizes per acetabular model type for the different stability tests are shown in Table 2.

### 3.2. Relative Micromotion Measurement

**Figure 7:**
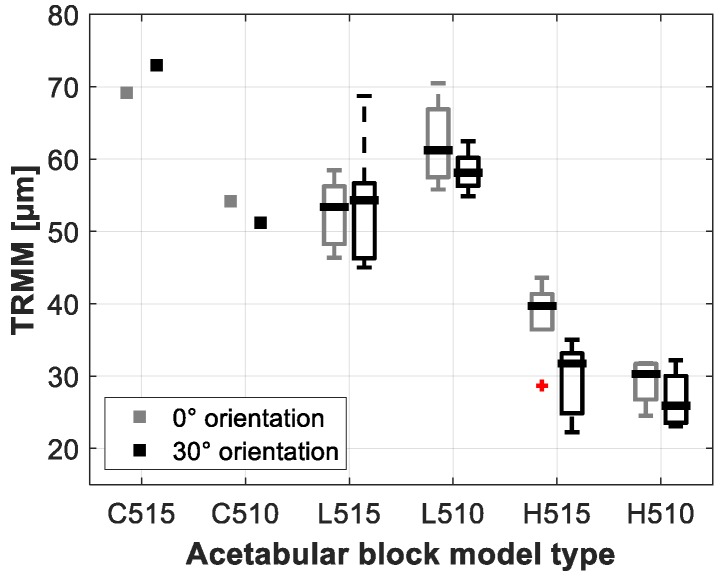
Boxplot representation of the total resulting micromotion (TRMM) measured under 0° and 30° loading orientations for the different acetabular models.

**Figure 8:**
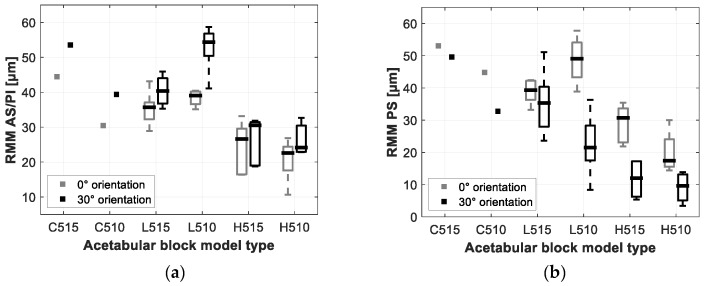
Boxplot representation of the resulting local micromotion (RMM) measured under 0° and 30° loading orientations for the different acetabular models. (**a**) shows the results measured at the AS/PI zone of the acetabular model, (**b**) shows the results measured at the PS zone of the acetabular model.

The total resulting micromotion (TRMM) and resulting local micromotion (RMM) measured for the different acetabular models are summarized in Figure 7 and Figure 8. Table 3 gives an overview of the results of the TRMM for the different acetabular models.

#### 3.2.1. Loading Orientation

The loading orientation did not result in any significant difference in TRMM (*p* = 0.068). The local RMM however, showed to be significantly higher under 30° loading at the AS/PI zone (*p* << 0.05) and significant lower under 30° loading at the PS zone (*p* << 0.05).

#### 3.2.2. Acetabular Model Density

Significant differences in the TRMM were observed between the low density acetabular models (0.48 g/cc) and the high density acetabular models (0.80 g/cc). The lower density models showed, on average, 56.7 µm TRMM versus 30.9 µm for the higher density models (p-values varied between 0.006 and 0.021 depending on the model orientation and press-fit).

#### 3.2.3. Press-Fit

The TRMM measured in the low density acetabular models under 0° orientation showed to be significantly lower when using a higher press-fit (52.7 µm for a 1.2 mm press-fit, 62.2 µm for a 0.7 mm press-fit (*p* = 0.033)). The other TRMM measurement resulted in no significant differences (*p*-Values varied between 0.086 and 0.462, depending on the acetabular model orientation and density).

### 3.3. Push-Out Experiment

**Figure 9:**
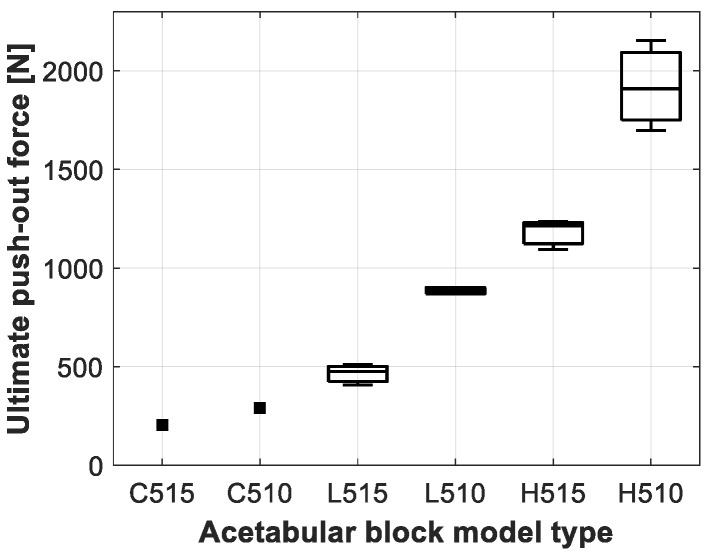
Boxplot representation of the ultimate push-out force measured for the different acetabular model types.

**Figure 10:**
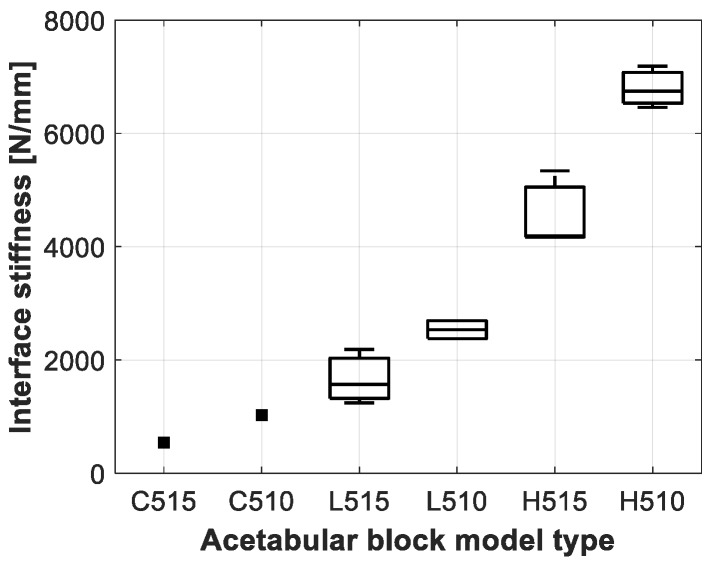
Boxplot representation of the interface stiffness measured for the different acetabular model types.

**Figure 11:**
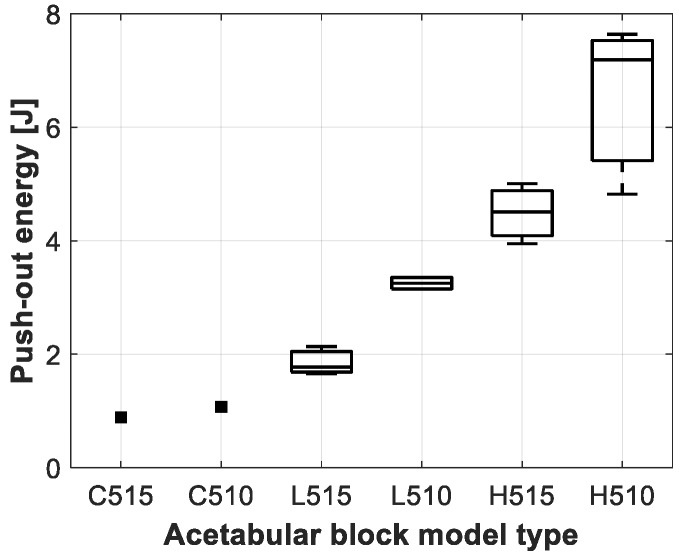
Boxplot representation of the push-out energy measured for the different acetabular model types.

The results of the push-out experiment for the different acetabular model types are presented in Table 4 and Figure 9, Figure 10 and Figure 11. Clear differences could be observed between the acetabular model types, but no significance could be obtained, due to the low sample size of the push-out tests as presented in Table 2.

### 3.4. Relation between Micromotions and Push-Out Experiment

**Figure 12:**
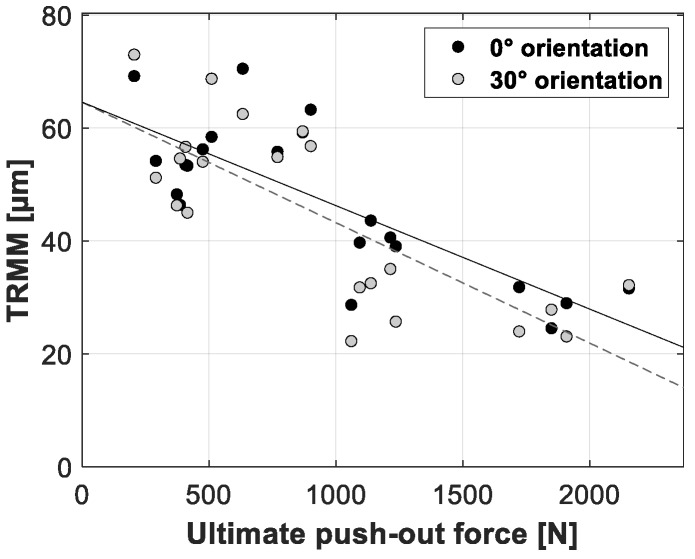
Scatter plot of the ultimate push-out force (*x*-axis) and the corresponding total resulting micromotion measured for each acetabular model (*y*-axis). The results for both loading orientations are shown in each plot (0° black dots, 30° gray dots). A linear function is fitted between both variables; the solid line represents the 0° loading orientation, and the dashed line represents the 30° loading orientation.

**Figure 13:**
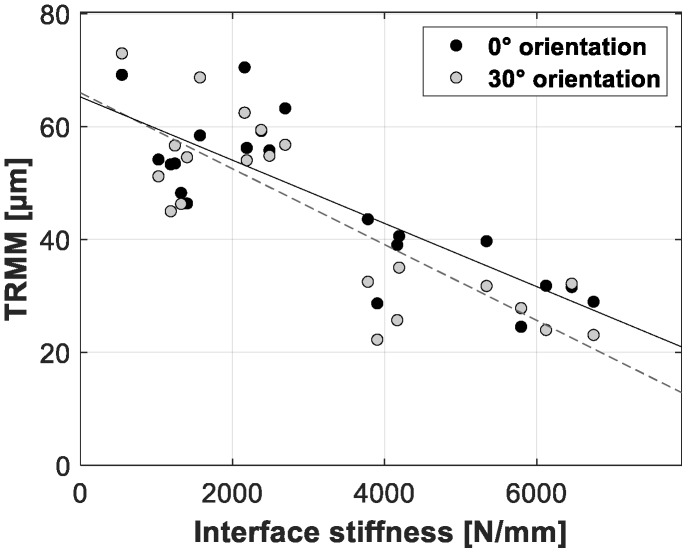
Scatter plot of the interface stiffness (*x*-axis) and the corresponding total resulting micromotion measured for each acetabular model (*y*-axis). The results for both loading orientations are shown in each plot (0° black dots, 30° gray dots). A linear function is fitted between both variables; the solid line represents the 0° loading orientation, and the dashed line represents the 30° loading orientation.

**Figure 14:**
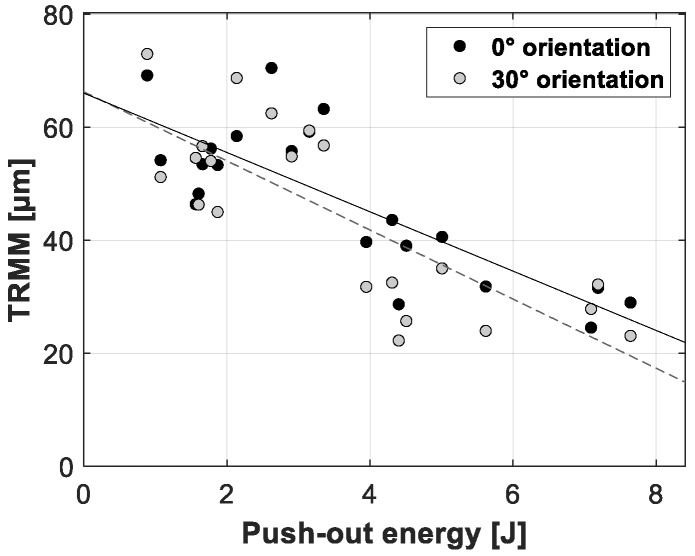
Scatter plot of the push-out energy (*x*-axis) and the corresponding total resulting micromotion measured for each acetabular model (*y*-axis). The results for both loading orientations are shown in each plot (0° black dots, 30° gray dots). A linear function is fitted between both variables; the solid line represents the 0° loading orientation, and the dashed line represents the 30° loading orientation.

**Figure 15:**
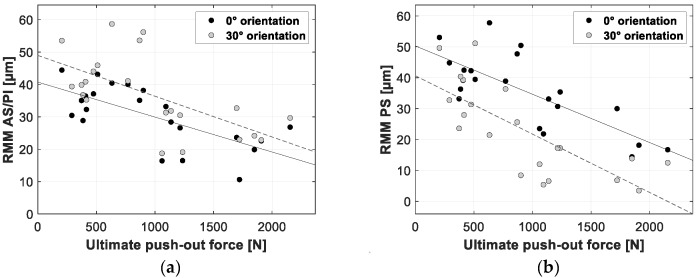
Scatter plots of the ultimate push-out force (*x*-axis) and the corresponding resulting micromotion measured at the AS/PI (**a**) and PS (**b**) zone of the acetabular model (*y*-axis). The results for both loading orientations are shown in each plot (0° black dots, 30° gray dots). A linear function is fitted between both variables; the solid line represents the 0° loading orientation, and the dashed line represents the 30° loading orientation.

**Figure 16:**
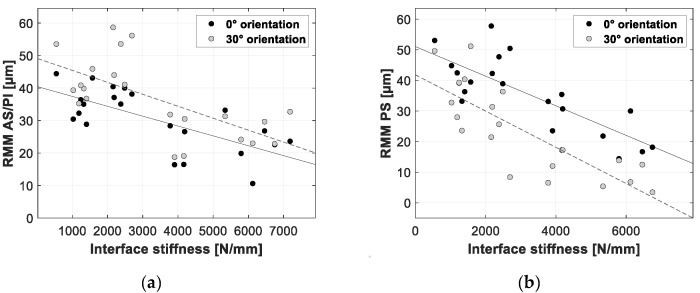
Scatter plots of the interface stiffness (*x*-axis) and the corresponding resulting micromotion measured at the AS/PI (**a**) and PS (**b**) zone of the acetabular model (*y*-axis). The results for both loading orientations are shown in each plot (0° black dots, 30° gray dots). A linear function is fitted between both variables; the solid line represents the 0° loading orientation, and the dashed line represents the 30° loading orientation.

**Figure 17:**
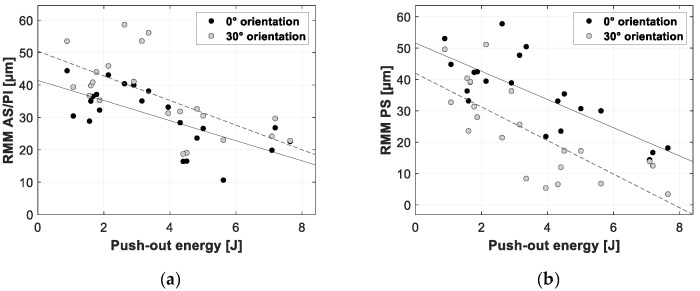
Scatter plots of the push-out energy (*x*-axis) and the corresponding resulting micromotion measured at the AS/PI (**a**) and PS (**b**) zone of the acetabular model (*y*-axis). The results for both loading orientations are shown in each plot (0° black dots, 30° gray dots). A linear function is fitted between both variables; the solid line represents the 0° loading orientation, and the dashed line represents the 30° loading orientation.

Figure 12, Figure 13 and Figure 14 present the linear regression between the TRMM and ultimate push-out force, interface stiffness and push-out energy, respectively. Table 5 presents an overview of the slope values and corresponding R^2^ values per combination of TRMM and push-out experiment stability metric matching the results presented in Figure 12, Figure 13, Figure 14, Figure 15, Figure 16 and Figure 17 present the linear regression between the local RMM and the ultimate push-out force, interface stiffness and push-out energy, respectively. Accordingly, Table 6 and Table 7 present an overview of the slope and the R^2^ values of the linear regression per combination of local RMM and push-out stability metric. All the stability metrics determined from the push-out experiment predicted the TRMM and local RMM significantly well (*p* << 0.05). Note that the R^2^ values of the linear regression of TRMM were consistently higher compared to those of the local RMM.

## 4. Discussion

The initial stability of the cementless acetabular implant is defined as the relative motion between implant and host bone under physiological loading immediately after implantation. This initial stability is one of the main factors in determining proper osseointegration of the implant over time [2]. In order to ensure proper osseointegration, no more than 150 µm relative motion between implant and bone should occur at the interface [3,4]. Measuring the relative motion between implant and bone in vitro requires a specific custom-made measurement set-up, which leads multiple studies to use alternative initial implant stability tests, such as push-out, lever-out, torsional loading or edge loading experiments which require fewer complex set-ups. The goal of this study was to assess the relationship between two different initial stability tests; the golden standard test of micromotion versus alternative load-to-failure implant stability metrics derived from a push-out experiment. Different stability metrics were derived from the push-out experiment similarly to ones that are used in previous research; ultimate push-out force [7,30,31], interface stiffness [15,18], push-out energy [21,32]. A push-out experiment was chosen over other load-to-failure tests, as the applied load direction during a push-out test is similar to a physiologic loading direction. An additional aim of this study was to investigate the influence of different bone material properties and press-fits on the stability of cementless acetabular implants.

Simplified replicate acetabular bone models were manufactured out of three different materials and using two different press-fits to mimic the acetabulum. This type of bone model is commonly used in the literature as an alternative to mimic the human acetabulum [6,7,14,15,17,21,22]. One rigid cellular polyurethane foam (0.32 g/cc density) and two types of solid rigid polyurethane foam (0.48 and 0.80 g/cc density) were tested using both a 0.7 mm and 1.2 mm press-fit. These three types of material were chosen to cover a broad range of healthy trabecular bone properties, which vary between 0.109 g/cc and 0.959 g/cc [33].

The presented results on TRMM measured with the low and high density acetabular models (ranging from 27 µm up to 62 µm) are in line with the range of micromotions of cementless acetabular cups documented by [7] who used similar artificial bone models and testing conditions. Crosnier et al. reported acetabular cup motions varying between 45 µm and 90 µm using similar densities and design of acetabular bone models and loading conditions (cyclic loading up to 2 kN, single leg stance). However, [7] did not take into account the bone motion as the implant motion was measured relative to the ‘ground’, which can clarify the somewhat higher micromotions compared to the results of this study as the bone is proved to behave non rigidly. Micromotions reported in cadaveric studies using cementless acetabular cups vary over a large range starting from 7.73 µm up to 162 µm [8,9,11,12,13]. It is not straightforward to compare the results of these cadaveric studies to the results presented in this study, as these cadaveric studies cover a wide variety of test variables, such as cadaveric bone properties, loading conditions, acetabular cavity preparation, implant positioning, etc. Despite the significant variety in cadaveric test variables, the results of this study lie within the range of measured micromotion reported in these cadaveric studies.

Reinserting the acetabular implant, a second time, in the acetabular model did not lead to significant differences in TRMM or local RMM. An extraction and reinsertion of the implant lead to minor wear of the acetabular cavity, which leads to a minor decrease in press-fit. This finding is in line with the lack of significant difference in TRMM and local RMM between the two tested press-fits. A possible explanation for this finding is that micromotion is related to the contact area between the implant and bone. This contact area is generated by the elastic deformation of the bone around the implant, due to the press-fit forces [2,17]. A certain amount of minimal press-fit (lower threshold) will correspond to a maximal contact area given the stiffness and geometry of the implant and acetabular cavity. A further increase of press-fit will not lead to a significant increase of contact area resulting in maximal and constant implant stability in terms of micromotion, which is seconded by the cadaveric study of [34] that reports a minor increase of contact area between implant and bone when comparing 1 mm and 2 mm press-fit. Other studies reported similar findings: An increase in press-fit did not result in a decrease of micromotion, while an exact fit (acetabular cavity diameter = acetabular cup diameter, 0 mm press-fit) mostly resulted in the highest amount of micromotion [8,9]. On the contrary, excessive press-fit can, on its turn lead to incomplete seating of the implant in the bone, especially in the case of stiff bone as reported by [17]. An additional study should confirm this concept of an optimal press-fit range in which micromotion tend to remain constant and minimal.

The loading orientation showed to have significant influence on the local RMM, as seen in Figure 1. The local RMM measured at the AS/PI location was significantly higher under 30° loading compared to 0° loading orientation, the opposite was seen at the PS measurement location. A logical explanation for this result can be found in the alignment between the measurement sensors and the load vector. Under 30° loading, the sensors at the AS/PI are more in parallel with the load vector, which is the opposite case at the PS measurement location. The TRMM was not significantly influenced by the loading orientation, indicating that the TRMM is an appropriate comparable global micromotion stability metric as it is not highly dependent on the local bone-implant behavior.

Bone density indicated to be a significant factor in implant stability. TRMM measured in the cellular bone models were on average, almost 9% (5 µm) higher than in the low density bone models (Table 1). TRMM measured in the low density bone models were almost 84% (26 µm) higher than in the high density bone models (Table 1), this difference proved to be significant given the tested sample size. When considering the push-out stability metrics, the cellular bone models showed on average 62.5% lower metric values than the low density models, the low density models showed on their turn on average 57.8% lower metric values than the high density models (Table 2). Similar findings are reported in the literature. [35] reported in an FE study that micromotion is highly and non-linearly affected by the bone stiffness. In vitro implant stability studies using comparable acetabular models reported similar findings. [21] showed increased edge loading stability when using higher density bone models. [7] indicated that higher density bone models resulted in lower micromotion compared to lower density bone models.

A high degree of correlation with little variation was obtained between the measured micromotion and the ultimate push-out force, interface stiffness and push-out energy. According to Table 5, Table 6 and Table 7 and Figure 12, Figure 13, Figure 14, Figure 15, Figure 16 and Figure 17, the push-out stability metrics proved to be better predictors of the TRMM than the local RMM. R^2^ values regarding the TRMM varied between 0.599 and 0.688, as presented in Table 5. The maximal correlation was consistently found between the TRMM and the interface stiffness, indicating that the interface stiffness derived from a push-out experiment is the preferable alternative stability metric. A possible explanation for this finding can be found in the definition of the interface stiffness: The interface stiffness is determined out of the part of the force-displacement curve where elastic deformation of the bone model occurs, as shown in Figure 6. This indicates that the interface stiffness is related to the combination of the bone elasticity and the contact area between the implant and bone. A similar relationship between the micromotion and the combination of bone elasticity and the contact area is previously described, which is a possible explanation of the maximal correlation between the interface stiffness and micromotion. The presented results show that load-to-failure tests are an appropriate tool to compare the implant stability between different specimen. However, care should be taken when interpreting load-to-failure metrics absolutely to accurately predict the implant stability without determining their relationship with the relative bone-implant micromotion.

A limitation of this study is the use of simplified replicate acetabular bone models. Previous studies used similar bone models [6,7,15,17,21]. Another study measured comparable micromotion in replicate and cadaveric femurs [36], demonstrating that artificial bone material is an effective substitute to simulate micromotion. These bone models have similar material properties as human trabecular bone and have the advantage of possessing very low interspecimen variability when machined in a controlled manner compared to cadaveric bones. This interspecimen variability is characteristic to that with cadaveric material even though these models are the closest representation of the in vivo setting. The range of micromotions measured in this study lies within the range reported from cadaveric studies, suggesting the suitability of the tested artificial acetabular bone models. Nevertheless, the presented micromotion measurement set-up forms an appropriate tool to isolate and compare different variables that could influence the implant stability (e.g., press-fit, bone material properties, implant design, acetabular cavity preparation technique, etc.). However, care needs to be taken when interpreting the absolute values of the micromotion measurements of set-ups using replicate bone models as these models are an approximation of the human acetabulum, consisting of different boundary conditions and bone morphology. Additional research to compare the implant stability measured in the presented acetabular models with artificial and cadaveric pelvis models using comparable experimental protocols is recommended to validate the use of these simplified replicate acetabular bone models for preclinical evaluation.

## 5. Conclusions

This study presents a compact mechanical set-up to test implant stability in artificial acetabular bone models. The set-up is developed to measure the local relative motion between implant and bone at different locations along the peripheral rim under physiological loading conditions. The use of the presented set-up can provide better insight during preclinical testing of new acetabular cup designs or new surgical procedures or provide a useful tool for surgical training.

Bone material properties have a major influence on implant stability. Denser and stiffer host bone results in increased initial stability of cementless acetabular cups. Care has to be taken when fixating a cementless acetabular cup in bone with lower stiffness and density (e.g., osteopenic bone). In this case, appropriate press-fit is important to maximize the bone-implant contact area and result in better implant stability. A high degree of correlation was found between micromotion measurements and stability metrics derived from push-out experiments. Hence, traditional load-to-failure tests can provide an adequate initial estimation of the implant stability without requiring a specific micromotion measurement set-up. Nevertheless, relative micromotion measurements between bone and implant remain the golden standard to quantify implant stability and are still preferred when highly accurate results are required.

## Figures and Tables

**Table 1:** Overview of the tested acetabular models; two single bone models (specimen 1 and 2) and four sets of three identical models (specimen 3–14).

Denotation	Density [g/cc]	Cavity Diameter [mm]	Specimen
C515	0.32	51.5	1
C510	0.32	51.0	2
L515	0.48	51.5	3-4-5
L510	0.48	51.0	6-7-8
H515	0.80	51.5	9-10-11
H510	0.80	51.0	12-13-14

**Table 2:** Sample sizes per acetabular model type for the different stability experiments.

Type	N Micromotion Experiment	N Push-Out Experiment
C515	1	1
C510	1	1
L515	6	3
L510	5 *	2 *
H515	5	3
H510	5	3

*: One acetabular model (specimen 6) was damaged during the micromotion measurement. Only the local RMM and TRMM under 30° loading could be measured for this specimen. No micromotion measurement under 0° orientation and push-out experiment was performed for this specimen.

**Table 3:** Overview of the mean TRMM and range [min, max] measured under 0° and 30° loading for the different acetabular models.

Type	TRMM 0° [µm] Mean [Min, Max]	TRMM 30° [µm] Mean [Min, Max]
C515	69 [–]	73 [–]
C510	54 [–]	51 [–]
L515	53 [46, 58]	54 [45, 69]
L510	62 [56, 71]	58 [55, 62]
H515	38 [29, 44]	29 [22, 35]
H510	29 [25, 32]	27 [23, 32]

**Table 4:** Overview of the mean and range [min, max] of the push-out metrics for the different acetabular model types.

Type	Ultimate Push-Out Force [N] Mean [Min, Max]	Interface Stiffness [N/mm] Mean [Min, Max]	Push-Out Energy [J] Mean [Min, Max]
C515	205 [–]	545 [–]	0.89 [–]
C510	290 [–]	1025 [–]	1.07 [–]
L515	464 [407, 510]	1666 [1242, 2186]	1.86 [1.66, 2.14]
L510	885 [869, 901]	2534 [2376, 2692]	3.25 [3.15, 3.35]
H515	1181 [1093, 1236]	4565 [4165, 5340]	4.49 [3.95, 5.01]
H510	1920 [1698, 2154]	6798 [6461, 7186]	6.55 [4.82, 7.64]

**Table 5:** Overview of the slope coefficient and R^2^ value of the linear regression analysis between the TRMM under 0° and 30° loading orientation, and the different push-out experiment stability metrics.

	Push-Out Experiment Stability Metric	Slope Coefficient	R^2^
**0°**			
	Ultimate push-out force	−0.018 µm/N	0.617
	Interface stiffness	−0.006 µm/N/mm	0.668
	Push-out energy	−5.250 µm/J	0.638
**30°**			
	Ultimate push-out force	−0.021 µm/N	0.599
	Interface stiffness	−0.007 µm/N/mm	0.688
	Push-out energy	−6.116 µm/J	0.620

**Table 6:** Overview of the slope coefficient and R^2^ value of the linear regression analysis between the local RMM at the AS/PI region under 0° and 30° loading orientation, and the different push-out experiment stability metrics.

	Push-Out Experiment Stability Metric	Slope Coefficient	R^2^
**0°**			
	Ultimate push-out force	−0.011 µm/N	0.480
	Interface stiffness	−0.003 µm/N/mm	0.486
	Push-out energy	−3.105 µm/J	0.471
**30°**			
	Ultimate push-out force	−0.013 µm/N	0.393
	Interface stiffness	−0.004 µm/N/mm	0.430
	Push-out energy	−3.802 µm/J	0.425

**Table 7:** Overview of the slope coefficient and R^2^ value of the linear regression analysis between the local RMM at the PS region under 0° and 30° loading orientation, and the different push-out experiment stability metrics.

	Push-Out Experiment Stability Metric	Slope Coefficient	R^2^
**0°**			
	Ultimate push-out force	−0.016 µm/N	0.572
	Interface stiffness	−0.005 µm/N/mm	0.634
	Push-out energy	−4.500 µm/J	0.597
**30°**			
	Ultimate push-out force	−0.019 µm/N	0.566
	Interface stiffness	−0.006 µm/N/mm	0.644
	Push-out energy	−5.367 µm/J	0.574
